# Comment on Kilian et al. Comparing Characteristics and Treatment of Brain Vascular Malformations in Children and Adults with HHT. *J. Clin. Med.* 2023, *12*, 2704

**DOI:** 10.3390/jcm12227179

**Published:** 2023-11-20

**Authors:** Omer F. Eker, Sophie Dupuis-Girod, Claire L. Shovlin, Edoardo Boccardi

**Affiliations:** 1 Hospices Civils de Lyon, 69006 Lyon, France; sophie.dupuis-girod@chu-lyon.fr; 2 CREATIS Laboratory, INSA-Lyon, 69621 Villeurbanne, France; 3 Imperial College London, London W12 0NN, UK; c.shovlin@imperial.ac.uk; 4 Imperial College Healthcare National Health Service Trust, London W12 0HS, UK; 5 Department of Neuroradiology, Grande Ospedale Maggiore Niguarda, 20162 Milan, Italy; dodibocc@yahoo.it

We read with interest the recent article by Killian et al. regarding the characteristics and treatment of brain vascular malformations (VMs) in children and adults with hereditary hemorrhagic telangiectasia (HHT) [1]. The authors described and compared the clinical presentations and the treatment approaches of HHT-related brain VMs in their pediatric and adult cohorts. The authors conclude that their “large data cohort supports the current recommendation for screening for brain VMs in pediatric patients and supports the case for systematic brain VM screening for adult patients with HHT”.

We respectfully disagree with their conclusions. We would like to point out several methodological points and conclusions that demand closer review.

First, the study reports an increased prevalence of intracranial hemorrhage (ICH) at presentation in HHT patients (up to 23.7% and 9.9% in children and adult populations, respectively). These data were extracted from a highly selected cohort and therefore a biased group of patients followed-up within the BRAIN consortium, which is not comparable to the previously reported data on larger-based HHT populations, ranging between 1% and 2% [2,3].

Second, the authors aimed to evaluate the characteristics of brain VMs in HHT patients without mentioning their types. The proportions of pial arteriovenous fistula (AVF), nidus-type arteriovenous malformation (AVM), capillary VM, and cavernous malformations were not specified. This information is essential to understand the population studied since these different VM types are associated with different risks of bleeding [4]. Indeed, the micro-AVMs (or capillary vascular malformations) are known to have a benign natural history, without any reports in the literature of ruptured cases in patients with HHT, whereas the nidus-type AVMs and pial arteriovenous fistulas (AVFs) are known to be at risk of rupture [5]. The rates of nidus-type AVMs and pial AVFs in the both children and adult groups of HHT patients with and without an ICH at presentation are essential to understand why such high bleeding rates were observed and which ones could have been avoided.

Third, the authors presented the proportions of treated brain VMs within the ruptured (23/27, 85%) and unruptured (46/87, 52.9%) groups of patients, respectively, rather than presenting the proportions of ruptured (23/69, 33.3%) and unruptured (46/69, 66.7%) VMs among the treated patients. In doing so, the results tend to obscure the high proportion of unruptured VMs treated in the pediatric population (up to 66.7%), without any certainty about their potential long-term risk of bleeding. Several previous studies that examined the natural history of nidus-type AVMs in HHT patients concluded an overall lower risk of bleeding of HHT-related nidus-type AVMs and that this does not advocate for their treatment if unruptured—references [2,6,7], including European interventional consensus supported by EANS, ESMINT, EGKS, and SINCH [8].

The fourth issue is with regard to missing justifications for treating the unruptured brain VMs in the study population. Justifications for brain VM treatment are important to understand the clinical practices in the center where the patients were admitted, and above all, are essential to justify any screening strategy and its consequences [9]. In [1], the clinical characteristics of HHT patients with brain VMs without any ICH at presentation included asymptomatic patients who were screened; patients with focal neurological deficit, headache, or seizure. The three latter presentations are not known to be associated to any potential risk of bleeding at long terms, and do not justify, in isolation, any therapeutic indication.

Fifth, only the incidence and the timing of post-treatment hemorrhage were reported without any details about the rates of: (1) the complete occlusion of the brain VMs, (2) post-therapeutic complications, also including brain necrosis after stereotactic radiotherapy [10], (3) the patient’s clinical outcome, and (4) the durations of follow-up. The cumulative incidence of post-treatment hemorrhage is reported according to the treatment modality, which brings no value to advocate for the screening strategy in HHT. It would have been more of an interest to report the cumulative incidence of post-treatment hemorrhage according to the presence of ICH at presentation, and the morbimortality rates of treated and untreated unruptured brain VMs in screened patients, to assess if the treatment brings benefit or harm to the patient. The lack of these endpoints of primary importance lessen the significance of the results, and certainly does not support the screening strategy for brain VMs in children with HHT.

These issues, in what were large but highly selected, and therefore biased cohorts of pediatric and adult HHT patients, consisting of incomplete, descriptive, and mixed results, have allowed the authors to draw and present erroneous conclusions. We are certain it was not their intention to misleadingly extrapolate from their data. However, instead of being “generalizable”, the results in [1] are impossible to apply to other patient groups, given unclear screening and justification criteria for treatment. Without evaluating the benefit and the risk of the screening and its consequences, the presented results do not demonstrate, nor even suggest, that the screening strategy and brain VM treatment reduces the risk of their complications in children with HHT.
